# Presence of HPV in prostate tissue from patients submitted to prostate biopsy

**DOI:** 10.1590/acb371205

**Published:** 2023-01-13

**Authors:** Nalisson Marques Pereira, Emerson Augusto Castilho Martins, Mateus Goes Quintela, Arthur Arantes da Cunha, Mauricio Moura dos Santos, Jaques Waisberg

**Affiliations:** 1 MSc, associate professor. Universidade Federal do Amapá – Department of Medicine – Macapá (AP), Brazil.; 2 PhD, associate professor. Universidade Federal do Amapá – Department of Medicine – Macapá (AP), Brazil.; 3 MSc. Universidade Federal do Amapá – Department of Medicine – Macapá (AP), Brazil.; 4 Graduate student. Universidade Federal do Amapá – Department of Medicine – Macapá (AP), Brazil.; 5 MD. Universidade Federal do Amapá – Department of Medicine – Macapá (AP), Brazil.; 6 PhD, full professor. Faculdade de Medicina do ABC – Department of Surgery – Santo André (SP), Brazil.

**Keywords:** Papillomavirus Infections, Prostatic Neoplasms, Real-Time Polymerase Chain Reaction, Image-Guided Biopsy

## Abstract

**Purpose:**

Prostate cancer (PCa) is the second most frequent cancer among men in the Western population. Infections, such as the one caused by the human papillomavirus (HPV), have been shown to promote inflammation that can lead to the appearance of neoplasms. This study aimed to verify the presence of HPV in neoplastic and non-neoplastic prostate tissue in patients undergoing prostate biopsy and its possible relationship with PCa.

**Methods:**

Prostate tissue fragments were collected by prostate biopsy and subjected to polymerase chain reaction with primers for the HPV L1 gene to identify the presence of the virus.

**Results:**

Among 162 patients, 10 (6.2%) had HPV and in 152 (93.8%) HPV was not identified in prostate biopsies. HPV was detected in 7/95 (7.4%) of patients with PCa, in 2/55 (3.6%) of patients without PCa, and in no patient with an inconclusive diagnosis of PCa. There was no significant difference (p = 0.487) of HPV presence in the tissue of patients with PCa.

**Conclusions:**

There were no significant levels of HPV L1 protein in prostate tissue. The findings suggest the absence of HPV oncogenic activity in the prostate tissue of patients with PCa.

## Introduction

Prostate cancer (PCa) is the second most common cancer among men in the Western Hemisphere population. About 1.276,105 new cases and 358,990 deaths from PCa were reported in 2018 worldwide^1^.

However, little is known about the exact mechanisms involved in the development of prostate cancer. Environmental and hereditary factors are assumed to play crucial roles in prostate carcinogenesis. Age, race, and family history are among these established risk factors for PCa. In addition, increasing epidemiological evidence, along with molecular research, suggests that viral infection would be a potential cofactor for the development of PCa^2^^-^ 4.

Nowadays, 17% of all cancers have the involvement of inflammation in their genesis^5^. Inflammation may induce and promote neoplasms by exposing tissues to highly reactive compounds and growth factors, and by promoting the increase of cell renewal^5^.

The current classification system for prostatitis includes four types: acute bacterial, chronic bacterial, chronic non-bacterial (inflammatory), and chronic pelvic pain syndrome^6^. It is plausible that prostatitis may be important in the etiology of PCa^6^.

The finding that infection with a specific subset of human viruses is the primary cause of a substantial fraction of human cancers is one of the most significant achievements in cancer etiology and intervention^7^^,^ 8. Recently, it has been estimated that virus infection is the central cause of more than 1.400,000 cancer cases annually, representing approximately 10% of the worldwide cancer burden^7^^,^ 8.

In 1964, the first human oncovirus was discovered, when Epstein-Barr virus (EBV) was detected in Burkitt lymphoma cells by electron microscopy^9^^-^ 11. HPV, one of the most important oncogenic viruses, is a protein envelope-less virus 52-55 nm in diameter belonging to the family Papillomaviridae. The genome consists of 7.200-8,000 base pairs (bp) of closed circular double-stranded DNA containing up to 10 open reading frames^12^.

The HPV genome comprises three functional regions: the long control region (LCR); the early region (E), that encodes the E1, E2, and E4-E8 genes, although the open reading frame of E8 and E5 are not present in the genome of all HPV types; and the late region (L), that encodes the L1 and L2 genes^1^^,^ 12.

Approximately 280 types of papillomaviruses have been described in vertebrates. Based on the genomic sequence of L1, the gene encoding the main capsid protein, more than 200 types of HPV infect humans and have been identified and characterized, with at least 14 high-risk types which may cause cancer^13^^,^ 14.

HPV is often transmitted through sexual activity. Oncogenic types of HPV, such as HPV 16/18, are associated with cervical cancer in women. Studies have also suggested possible links between HPV infections and other female cancers, such as anus, vulvar, vaginal, and breast cancers. In addition, HPV has also been shown to be an important risk factor for male anogenital and urinary cancers, such as of the penis, anus, and bladder^2^^,^ 15 ^,^ 16.

Except for age, ethnic origin, and family history of prostate cancer, which are well-established non-modifiable risk factors, the etiology of prostate cancer remains largely unknown^17^. The accumulation of epidemiological, biological, genetic, and experimental evidence has suggested that chronic inflammation may be associated with the initiation or progression of several types of cancer, including prostate cancer^17^.

In 1990, McNicol and Dodd reported that HPV types 16 and 18 are present in normal and cancerous human prostate tissues^2^^,^ 18. Since then, an increasing number of studies have detected high-risk HPVs in prostate carcinoma tissues by Southern Blot and/or polymerase chain reaction (PCR) analysis^2^^,^ 18. Penile and urethral HPV lesions have been described, as well as an increased risk of prostate cancer associated with sexual activity^19^^,^ 20.

The aim of this study was to evaluate the presence of HPV in prostate tissue in patients undergoing transrectal ultrasound-guided prostate biopsy and its possible relationship with prostate cancer.

## Methods

This research project was analyzed and approved by the Research Ethics Committees of the institutions involved in the study, Universidade Federal de São Paulo (number 2.726.521), and later the Universidade Federal do Amapá (number 2.093.140).

A prospective analytical study was carried out with 162 prostate tissue samples that were obtained from patients undergoing screening for PCa by transrectal ultrasound (USG)-guided biopsy, collected at Instituto de Oncologia e Mastologia de Macapá (Macapá, AP, Brazil) from December 2018 to January 2021.

Patients from the public and private healthcare network from the city of Macapá, seen by local urologists, were included in the study, where, after evaluation by digital examination of the prostate or elevation of the prostate specific antigen (PSA), prostate biopsy guided by transrectal USG was indicated.

Patients who refused to sign the informed consent form or who did not comply with the indication for prostate biopsy according to the international guidelines, *i.e.*, who did not present altered PSA levels or altered prostate digital examination, were excluded from the study^21^^,^ 22.

### Sample collection

Transrectal USG-guided biopsies collected 13 prostate tissue fragments from each patient, which all were included in the study. One of these fragments was collected from the right prostate base or from an area sonographically suspicious for an oncologic process.

This fragment was placed in a previously identified 1.5 mL microcentrifuge tube with 1 mL of RNAlater (Ambion, Thermo Fischer Scientific, Belford, MA, United States of America). After collection, the vials were stored at -20 °C until being processed.

### Pathological examination and DNA extraction

The pathological analyses were performed separately by three pathologists. The prostatic tissue staining was performed by the hematoxylin and eosin (HE) technique. The pathological examination identified patients with or without PCa, and patients with atypical small acinar proliferation (ASAP). The latter ones were submitted to immunohistochemical study to confirm or exclude malignancy in the tissue.

Total DNA extraction was performed in the Microbiology and Immunology Laboratory of the Universidade Federal do Amapá using 7-mm inox microbead in a Tissuelyzer LT (Quiagen^®^, Germany) for cell lysis (50 Hz, 15 min) with Trizol (Invitrogen^®^ Thermo Fischer Scientific, Belford, MA, United States of America), following the manufacturer’s instructions. To assess the concentration and purity of the extracted DNA, the samples were quantified by spectrophotometry (NanoDrop^™^ Onec Microvolume UV-Vis Spectrophotometer, Thermo Scientific, Belford, MA, United States of America). The A260 nm/A280 nmratio was evaluated by the equipment, in which the absorbance of DNA is captured at 260 nm and possible protein contamination is captured at 280 nm. Thus, samples with a ratio between 1.8 and 2 and with a minimum concentration of 10 ng/μL were considered viable for molecular analyses, totaling 162 samples used in the study.

### Real-time quantitative polymerase chain reaction

The real-time quantitative PCR (RT-qPCR) method (Real-Time PCR System 7500, Applied Biosystem Inc^®^, Carlsbad, CA, United States of America) was used for HPV detection. The test was set up in duplicate for each sample using the GP5+/6+ primer set (F 5’ TTTGTTACTGTGGTAGATACTAC3’ and R 5’ GAAAAATAAACTGTAAATCATATT 3’) that amplifies a 150-bp fragment from a conserved region of the L1 gene for HPV 16, 18, 56, 59 and 66.

The primers were previously tested using DNA extracted from cell lines contaminated with HPV (SiHa and HeLa). A positive control with a 1:1,000-dilution of DNA was used as a positive control of the reactions, simultaneously with the diagnosis of the presence of HPV in the samples. As DNA extraction control, the human glyceraldehyde 3-phosphate dehydrogenase (GAPDH) gene was used as target with the use of the primer pair of the same name (F 5’ CGAGATCCCTCCAAAATCAA3’ and R 5’ GATGGAGTTGAAGGTAGTTTCGTG 3’) that amplifies a 294 bp fragment. The reagent used for fluorescence generation was Power SYBR Green 2x (Applied Biosystems Inc^®^, Carlsbad, CA, United States of America) and amplification occurred for 45 cycles with 94 °C for 20 s to denaturation and 1 min at 60 °C for annealing/extension. The complete RT-qPCR protocol is described in Table 1.

**Table 1:** Real-time quantitative polymerase chain reaction protocol.

Reagent	Concentration (nM)	Volume/1 reaction (μL)
Sybr GREEN 2X	-	6.25
Primerforward	10	0.38
Reverse primer	10	0.38
H_2_O	-	2.4
DNA	-	2.5
Final volume		12

### Statistical analysis

The inferential analysis employed to investigate the relationship between the presence of HPV and cancer was the extension of Fisher’s exact test^23^.

In all conclusions obtained by the inferential analyses, the alpha significance level equal to 5% was applied. Statistical Package for the Social Sciences (SPSS) Statistics version 24 (IBM Inc., Armonk, NY, United States of America) was used as the statistical analysis program.

## Results

The sample selected in this research was composed of 162 patients, among who 10 (6.2%) had HPV present in the prostate tissue, and in 152 (93.8%) patients HPV was not identified.

The mean age of the patients was 67.9 years old (43 to 93). The mean PSA level was 10.7 ng/dL (1.3 to 4,664).

The biopsy results of the 162 patients confirmed cancer in 86 (53.1%), excluded cancer in 43 (26.5%), and in 33 (20.4%) the diagnosis was inconclusive. Of the latter, nine patients had cancer confirmed by immunohistochemistry examination, in 12 the diagnosis of cancer was excluded, seven patients had an ASAP classification, and for the remaining five patients the results of immunohistochemistry examinations were not performed by them.

Considering the biopsy and immunohistochemistry results conjointly, it was possible to classify 157 patients, 95 (60.5%) with PCa, 55 (35%) without cancer, and seven (4.5%) as inconclusive (ASAP).

The presence of HPV and the PCa relation are shown in Table 2. In all diagnoses, the presence of HPV was 7/95 (7.4%) in patients with cancer, and 2/55 (3.6%) in those without evidence of cancer or inconclusive diagnosis (ASAP). The inferential results revealed that the presence of HPV was not related to the diagnosis of cancer when the inconclusive (ASAP) diagnosis was considered in the analysis (p = 0.663) and also when the inconclusive (ASAP) diagnosis was not included (p = 0.487).

**Table 2:** Distribution of HPV presence among patients, according to cancer diagnosis (biopsy/immunohistochemistry).

Cancer (confirmed by biopsy/Immunohistochemistry)														
HPV	Yes			No			Inconclusive (ASAP)			Total	
Yes	7	7.4%		2	3.6%		-	-		9	5.7%
No	88	92.6%		53	96.4%		7	100%		148	94.3%
Total	95	100%		55	100%		7	100%		157	100%

HPV: human papillomavirus; ASAP: atypical small acinar proliferation.

## Discussion

PCa is one of the leading causes of neoplastic disease among men, with increasing mortality levels year after year, reaching nearly 400,000 deaths annually in recent years^1^.

The incidence of PCa is remarkable for its substantial global variation. There is a difference that can increase the incidence 40-fold when adjusted for age. This difference in incidence is also found when adjusted for geographic location, with its incidence being highest in African American men in the United States of America and lowest in Asian men living in their home countries^24^.

Langston *et al*.^25^ observed positive associations between clinical prostatitis and PCa, which would support the role of prostate inflammation in cancer development. However, there is a question regarding the influence of inflammation on the onset of PCa, as no study has considered the potential for detection bias in their pooled estimates. These biases occur because men with clinical prostatitis are more likely to be screened or investigated for PCa than men without prostatitis^25^.

Although most HPV infections do not cause symptoms and resolve spontaneously, persistent infection with oncogenic HPV types, also known as high-risk HPVs, may lead to the appearance of precancerous lesions and cancer^15^^,^ 16. High-risk HPVs are not only responsible for virtually all cases of cervical cancer, but are also causally related to a variable fraction of other anogenital cancers (vulvar, vaginal, penile, and anal) and a subset of head and neck cancers, particularly of the base of the tongue, tonsil, and other oropharyngeal cancer sites^15^^,^ 16.

A potential etiological factor of interest for PCa is the exposure to HPV. The prostate may be a target of HPV infection for anatomical reasons, mainly by direct access of viral particles through the urethra^19^. The current literature on the possible role of HPV infection in PCa carcinogenesis remains controversial^1^^,^ 2.

The present study had an initial sample of 180 patients, and after the stratification of the data collected, 162 patients were selected. Most studies employed tissues that were products of prostate surgeries, *i.e.*, the patient’s previous disease was already known. These tissues were mostly formalin-fixed and paraffin-embedded. Zambrano *et al*.^26^ found that tissue fixation and preservation procedures damage DNA and affect the subsequent analysis of the RT-qPCR reaction. Several factors, such as the amount of time the tissue is stored prior to fixation, the number of hours the tissue was immersed in the fixing agent, and the age of the tissue blocks used may influence DNA integrity and could explain divergent results from different laboratories.

The current study used USG-guided transrectal collection of prostate tissue to minimize contamination. Most studies used surgical specimens, and, in a single study, biopsies were performed under ultrasound guidance, but by transperineal approach, a region with a high incidence of HPV^27^^,^ 28.

However, fresh frozen tissue may better preserve DNA^26^. Sadri Nahand *et al*.^1^ used tissue samples stored at -80 °C immediately after being frozen. The results revealed the presence of HPV in 15% of benign prostatic tissue and in 32% of PCa tissue.

Our samples were collected and placed in an RNA stabilization reagent (RNAlater) and, then, were immediately stored at low temperatures to obtain the best possible quality of genetic material and to reduce bias in the quality of the material.

Laprise *et al*.^29^ showed that the presence of HPV in semen was estimated in 16% of selected men in fertility clinics (under evaluation and/or treatment) and in 10% of men in the general population. These values are considered low to correlate with a transmission. Other studies have also shown a low prevalence of HPV in semen. Moghimi *et al*.^30^ studied 140 men who had their semen evaluated. Only eight (11.4%) were positive for HPV. Tuominen *et al*.^31^ found 19% HPV in their semen samples, and only one-third of the HPV-positive patients had high oncologic risk subtypes.

Dunne *et al*.^32^ performed a systematic review and concluded that urine, semen, and urethral swabs are the least useful specimens due to secondary infection. The source of infection in these specimens is unclear and may come from the urethra, seminal fluid, or the ductus deferens. Semen and urethral samples may contain more human β-globin and HPV DNA, but they are difficult and sometimes uncomfortable to collect. Coamplification of a human cellular target, in this case, human β-globin is one way to confirm human DNA in a sample^33^.

The detection of β-globin and HPV DNA is generally not effective in urine samples, although these samples may be the easiest to obtain. These data reflect the difficulty of HPV to reach the prostate^32^.

The present study sought to clarify whether HPV may be involved in PCa emergence, which is extremely relevant, since vaccines against HPV are available to provide excellent prevention and control of this viral infection and its possible consequences, such as the development of tumors.

After carrying out the real-time PCR test with primers for the 150 bp HPV L1 gene, we found that 6.2% of samples were positive for HPV, which was compatible with what was observed by Balis *et al*.^33^. The authors used the conventional PCR test and found 4.8% of HPV in their samples, but no strains with oncogenic potential, including HPV 16 and 18.

A similar study was performed by Silvestre *et al*.^34^ with the Linear Array HPV Genotyping Test (Roche Molecular Diagnostic, Alameda, CA, United States of America), who found 3% HPV in the samples analyzed, all of HPV 16. The Linear Array test uses the PGMY09/11 L1 consensus primer system to amplify a 450 bp HPV fragment and includes human β-globin as a control for DNA viability.

Araujo-Neto *et al*.^35^ and Sfanos *et al*.^36^ used conventional PCR as a form of DNA extraction and did not find HPV in PCa tissue samples in their studies.

In contrast, Medel-Flores *et al*.^37^ observed 14.9% HPV positivity in PCa using PCR with primers for the 150 bp HPV L1 gene. Atashafrooz *et al*.^38^ using a similar technique as in the present study found 14% positivity for HPV in PCa.

In the present research, we tried to identify the possible biases of published studies regarding the identification of oncogenic HPV in prostate tissue. Hence, we did not use paraffin-embedded or formalin-embedded tissue, which is posed as a limiting factor for the quality of the stored DNA. We employed fresh tissue with RNA stabilizer and freezing, whereas almost all studies with fresh prostate tissue used only low temperature as a source of preservation.

One factor that may account for the 6.2% HPV positivity found in our study is the fact that the rectum is a site in which HPV has already been found, and, since the collection was carried out by using a transrectal transducer, it is possible that the tissue may have been contaminated with HPV of rectal origin^39^^-^ 41.

## Conclusion

There was neither L1 gene expression nor significant levels of HPV L1 in non-neoplastic and neoplastic prostate tissue. These findings suggest no oncogenic activity of HPV in PCa.
